# Novel pathway to produce high molecular weight kraft lignin–acrylic acid polymers in acidic suspension systems†

**DOI:** 10.1039/c7ra12971h

**Published:** 2018-03-29

**Authors:** Fangong Kong, Shoujuan Wang, Weijue Gao, Pedram Fatehi

**Affiliations:** Key Laboratory of Pulp and Paper Science and Technology of Ministry of Education, China, Qilu University of Technology Jinan 250353 China; Chemical Engineering Department, Lakehead University Thunder Bay ON P7B 5E1 Canada pfatehi@lakeheadu.ca nancy5921@163.com +1-807-343-8697

## Abstract

Kraft lignin (KL) produced in kraft pulping process has a low molecular weight and solubility, which limits its application in industry. For the first time, KL was polymerized with acrylic acid (AA) in an acidic aqueous suspension system to produce a water soluble lignin–AA polymer with a high molecular weight in this work. The polymerization reaction was carried out using K_2_S_2_O_8_ as an initiator, and the influence of reaction conditions on the carboxylate group content and molecular weight of resultant lignin polymers was systematically investigated. The mechanism of polymerization of KL and AA was discussed fundamentally. The resulting lignin–AA polymer was characterized by Fourier Transform Infrared spectrophotometry (FTIR), proton nuclear magnetic resonance (^1^H-NMR) and elemental analyses. The results showed that the phenolic hydroxyl group (Ph-OH) content of KL promoted the polymerization under an acidic environment. Under the conditions of 1.5 wt% of initiator, 3.5 of pH, 10.0 of AA/lignin molar ratio, 0.15 mol L^−1^ of lignin concentration, 3 h and 80 °C, the carboxylate group content and the molecular weight of the polymer were 7.37 mmol g^−1^ and 7.4 × 10^5^ g mol^−1^, respectively. The lignin–AA polymer was water soluble at a 10 g L^−1^ concentration and a pH higher than 4.5. Furthermore, the flocculation performance of lignin–AA polymer in an aluminium oxide suspension was evaluated. Compared with polyAA, the lignin–AA polymer was a more efficient flocculant for aluminium oxide suspension, which shows its potential to be used as a green flocculant in industry.

## Abstract 2

Kraft lignin (KL) produced in kraft pulping process was polymerized with acrylic acid in an aqueous system.

## Graphical abstract

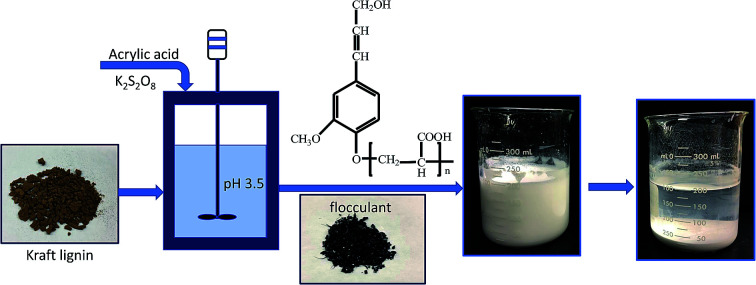

## Introduction

Lignin is a natural biomacromolecule, found in wood and vascular plants. Over 60 million tons of lignin is produced in the pulp and paper industry annually in the world.^1^ Among technical lignins produced, kraft lignin is the most dominant one, but is mainly incinerated as a low cost fuel in the pulping industry leading to the waste of resources and growing environmental problems.^2,3^ With the depletion of fossil fuel and the improvement of environmental awareness, greater endeavors have been made on developing lignin-based materials. However, kraft lignin has not yet been utilized effectively.^4,5^

Lignin is a highly stable and complex compound with a three-dimensional aromatic structure formed from three phenylpropanoid monomer units of guaiacyl, syringyl and *p*-hydroxyphenyl, connected by ether and carbon–carbon bond in an irregular form.^6^ Various modification techniques were carried out in the past to produce new lignin-based products for beneficial purposes.^7^ One of these modifications is the polymerization of lignin with functional monomers, which can increase both the molecular weight and number of functional groups on lignin structure. Chen *et al.*^8^ produced a polymer by reacting lignosulfonate with 1-ethenylbenzene to enhance thermal stability and molecular weight of the polymer. The polymerization of lignosulfonate with vinyl monomers, *i.e.*, acrylic acid, acrylonitrile, and methyl methacrylate, was also studied in aqueous or organic solvents in the past *via* chemical radical starters^9–12^ or chemo-enzymatic starters,^13^ in order to produce high molecular weight products with high hydrophilic or hydrophobic properties. Meister *et al.*^14,15^ synthesized acrylamide with kraft lignin in dioxane solution and used it as a drilling mud additive. In another report, 1-phenylethylene–kraft lignin polymer was produced in dimethyl sulfoxide solution and used as an oil recovery agent. However, organic solvents, such as dioxane and dimethyl sulfoxide, were generally used for facilitating the homogeneous polymerization of kraft lignin and other monomers. However, these solvents are usually toxic, expensive and may need a complex recovery process after use, which hampers their practical application in industry.

In the past, the polymerization of lignin in aqueous solutions was assessed. Ibrahim *et al.*^16^ polymerized soda lignin with 2-acrylamido-2-methylpropane in 1 wt% NaOH solution, and the product, soda lignin polymer with a molecular weight of 2.6 × 10^6^ g mol^−1^, was used as a drilling mud additive. Fang *et al.*^17^ also produced a corn-stalk lignin–acrylamide polymer in NaOH solution and used it as an adsorbent for dye removal from wastewater. However, there is no report about the polymerization of kraft lignin and acrylic acid in aqueous acidic suspensions, which is studied for the first time in this work.

As proven in the literature, the complex and heterogeneous structure of lignin played important roles in its polymerization.^10,12,17–19^ However, there are contradictory reports on the role of phenolic group in the polymerization of lignin: (1) the phenolic group acts as an inhibitor owing to the quinoid structure produced in the polymerization, which was observed in the polymerization of styrene with lignosulfonate^18^ or with hydrochloric softwood lignin;^19^ (2) the phenolic group acts as an active centre for the polymerization. It was observed that the conversion rates of acrylic monomers, *i.e.*, AA, acrylonitrile and methyl methacrylate, were significantly accelerated in the presence of lignosulfonate.^4,11^ Therefore, the role of phenolic group on the polymerization of KL and AA is still not clear, but is crucial for understanding the polymerization of KL and AA from academic and industrial points of views. In the polymerization reaction, the aliphatic hydroxyl groups of KL might react with carboxylate group of poly acrylic acid (PAA) formed during the lignin–AA polymerization reaction through esterification, which would also graft PAA onto lignin.^20^ It is not clear if the esterification reaction would happen in an acidic heterogeneous condition, which is the second objective of this study.

In the work presented herein, the polymerization of kraft lignin with acrylic acid in an acidic aqueous solution was conducted using K_2_S_2_O_8_ as an initiator. The main aim of this study was to generate water soluble lignin–AA polymer with a high molecular weight, which will facilitate its application as a flocculant in wastewater systems. In addition, the influence of phenolic group on the polymerization efficiency was identified. This study also intended to report how the functional groups and molecular weight of softwood kraft lignin will be affected by this polymerization. The properties of the lignin–AA polymer were determined using a light laser scattering technique, and the flocculation performance of the resulting lignin–AA polymer for an aluminium oxide suspension, as a model suspension system for representing wastes of the mining industry, was evaluated by a photometric dispersion analyzer.

## Experimental

### Materials

Softwood kraft lignin sample with a molecular weight (*M*_w_) of 17 890 g mol^−1^ was received from FPInnovations' pilot plant facilities in Thunder Bay, ON. The kraft lignin was produced *via* LignoForce™ technology.^20^ Polydiallyldimethyl-ammonium chloride (PDADMAC, 100 000–200 000 g mol^−1^), acrylic acid (AA), potassium persulfate (K_2_S_2_O_8_) (analytical grades), sodium hydroxide (97%, reagent grade), hydrochloric acid (37%, reagent grade), potassium hydroxide (8 mol L^−1^ solution), potassium permanganate (analytical grades), ferrous ammonium sulfate (analytical grades), 0.1 mol L^−1^ hydrochloric acid, D_2_O, trimethylsilyl propanoic acid (TMSP), dimethyl sulphate and *para*-hydroxybenzoic acid (analytical grades) were obtained from Sigma-Aldrich Company, and used as received. Dialysis membrane (cut off of 1000 g mol^−1^) was obtained from Spectrum Labs.

#### Polymerization of KL with AA

The reactions were carried out in a nitrogen atmosphere in 250 mL three-necked flasks equipped with magnetic stirrers. At first, 2 g of lignin was suspended in 30 mL of deionized water at room temperature and 300 rpm for 20 min in three neck flasks. After that, the required amount of AA was added to the flasks and the final pH of the suspensions was adjusted to 3.5, using 1.0 mol L^−1^ NaOH solution. Subsequently, the temperature of the flasks was adjusted by keeping the flasks in a water bath and the solutions were purged with nitrogen for 20 min. Afterwards, the predetermined amount of initiator (K_2_S_2_O_8_, wt% based on lignin's weight) was added to the flasks in order to initiate the reaction at 300 rpm. A continuous supply of nitrogen was maintained throughout the reaction. The polymerization reaction was repeated under different temperatures (60 °C, 70 °C, 80 °C, 90 °C and 95 °C), time intervals (0.5 h, 1 h, 2 h, 3 h, 4 h and 5 h), initiator dosages (0.5 wt%, 1.0 wt%, 1.5 wt%, 2.0 wt%, 2.5 wt% and 3.0 wt%, based on lignin's weight), AA to lignin molar ratios (1.35, 2.70, 5.4, 8.0, 10, 13.5 and 16.3) and lignin concentrations (0.07 mol L^−1^, 0.1 mol L^−1^, 0.15 mol L^−1^, 0.22 mol L^−1^ and 0.38 mol L^−1^) in order to optimize the reaction conditions.

#### Extraction of lignin–AA polymer from reaction media

The purification of lignin–AA polymer was carried out according to the procedure developed in our earlier paper.^21^ The reaction solution was firstly acidified to pH of 1.5 to precipitate the lignin–AA polymer from the solution, and then centrifuged to remove the formed PAA homopolymer and unreacted AA monomer from lignin–AA polymer. The detailed procedure was stated in the ESI.

#### PAA preparation

PAA was prepared in the presence of KL under the conditions of pH 3.5, 0.15 mol L^−1^ lignin concentration, 8 AA/lignin molar ratio, 80 °C, 3 h and 1.5 wt% initiator. In the absence of KL, PAA was also produced under the same conditions. After the reactions, the solution was treated according to the procedure used in our earlier paper^21^ (available in ESI†).

#### Reaction of KL with PAA

In one set of experiments, the PAA, produced in AA system, was reacted with KL under the conditions of 0.15 mol L^−1^ of lignin, 8.0 mol mol^−1^ of PAA/KL, 1.5% initiator, 80 °C, 3 h and pH 3.5. After the reaction, the solution was purified as stated above and the final product was used for H-NMR analysis.

#### AA conversion analysis

The AA conversion in the reactions was determined using H-NMR. In this set of experiments, 0.2 mL of reaction solution was collected and then mixed with 0.8 mL D_2_O containing 5 mg mL^−1^ trimethylsilyl propanoic acid (TMSP) as an internal reference. The NMR spectra of these samples were recorded using an INOVA-500 MHz instrument (Varian, USA) with a 45° pulse and relaxation delay time of 1.0 s. The area of the peak at 5.95–6.05 ppm was considered for determining the unreacted AA concentration, *C*_1_, in the reaction solution. The AA conversion was calculated using eqn (1)1
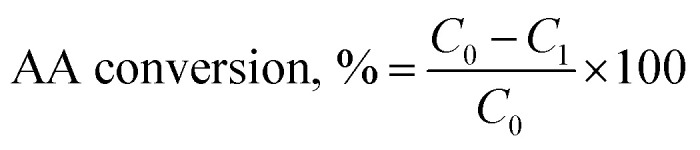
where *C*_0_ was the initial AA concentration in reaction solution, mol L^−1^ and *C*_1_ was the unreacted AA concentration in reaction solution, mol L^−1^.

#### Analysis of initiator consumption

A set of reaction was carried under the conditions of at 80 °C for 3 hours in a nitrogen atmosphere in 250 mL three-necked flasks, but without AA. At first, 25 mg of lignin was suspended in 50 mL of deionized water. Afterward, the initiator was added to the flasks to make an initial K_2_S_2_O_8_ concentration of 0.75 g L^−1^. The final pH of the suspensions was adjusted to 3.5 or 10.5 using 1.0 mol L^−1^ NaOH and HCl solutions. A continuous supply of nitrogen was maintained throughout the reaction. Similar reactions were carried out only with K_2_S_2_O_8_ at the same pHs (*i.e.*, control samples). The concentrations of initiator under acidic and alkaline conditions before and after the reactions were analysed by back titration with standardized potassium permanganate with a standardized ferrous ammonium sulfate solution.^22^ 10 mL of persulfate containing solution was pipetted to a 100 mL Erlenmeyer flask. Then, 10 mL of 0.5 M H_2_SO_4_ solution and 10 mL of 0.025 M ferrous ammonium sulfate solution were added to the flask while stirring constantly. After one min of stirring, the solution was titrated against 0.002 M KMnO_4_ to a permanent pink endpoint. A blank titration was conducted on 10 mL of ferrous ammonium sulfate solution in 10 mL of the 0.5 M H_2_SO_4_. The concentration of persulfate in the solution was calculated based on eqn (2)2
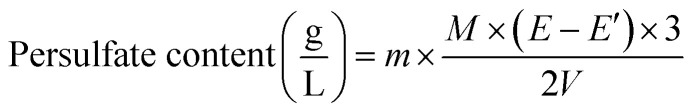
where *E* is the volumes (L) of KMnO_4_ solution used for the endpoint for blank sample. *E*′ is the volumes (L) of KMnO_4_ solution used for the endpoint for actual sample, *M* is the molarity (mol L^−1^) of KMnO_4_, *V* is the volume (L) of the sample and *m* is the molar mass of K_2_S_2_O_8_.

#### Acetylation of lignin

In order to understand if AA can react with other reaction sites on KL (in addition to phenolic hydroxyl groups) under acidic conditions, the acetylation of lignin was carried out according to the method described by Andes *et al.*^22^ In this set of experiments, 0.5 g of KL was dissolved in 6 mL of pyridine–acetic anhydride (1/1, v/v) by stirring for 30 min at 300 rpm, 25 °C and then kept in the dark at room temperature for 72 h. The solution was added drop-wisely to 120 mL of cold water and then centrifuged and washed 3 times. The solvent was removed from the sample using a freeze dryer and the final product was considered as acetylated lignin sample.

#### Methylation of KL

In order to understand the relationship between Ph-OH group content of KL and polymerization, KL was methylated according to the method reported in the literature.^23^ The reaction scheme is available in ESI (Fig. S1†). In this method, only Ph-OH group of KL can be methylated, while the aliphatic hydroxyl group of KL is methylated marginally.^23^ A 1.0 g of KL was dissolved in 15 mL of 0.7 mol L^−1^ NaOH solution at room temperature by stirring at 300 rpm for 20 min. After that, 0.25 mmol, 0.50 mmol or 1.0 mmol of dimethyl sulphate was added per each mmol of total phenolic hydroxyl groups of KL, and the solution was stirred at room temperature for 30 min. The solution was then heated to 80 °C for 2 h. During the reaction, the pH of the solution was kept at 11–11.5 by continuous addition of 0.7 mol L^−1^ NaOH solution. Upon the completion of reaction, the solution was acidified to pH 2.5 using 2 mol L^−1^ HCl solution and precipitates were washed with excess amount of deionized water until neutral pH was obtained and then the precipitates were freeze-dried. The final product was considered as methylated KL. The methylation conditions and the content of phenolic hydroxyl group of methylated KLs were listed in Table 1.

**Table 1:** Methylation conditions and Ph-OH content of methylated lignin

Sample	KL	1	2	3
Dimethyl sulphate/phenolic group of KL, mol mol^−1^	0	0.25	0.50	1.0
Ph-OH group, mmol g^−1^ lignin	1.73	1.41	1.02	0.70
Carboxylate group,mmol g^−1^ lignin	0.37	0.38	0.36	0.36

#### H_2_O_2_ treatment of kraft lignin

In order to further testify the influence of Ph-OH content of KL on the polymerization efficiency in the presence of carboxylate groups, hydrogen peroxide was used to treat KL to prepare the treated kraft lignin samples with different Ph-OH and carboxylate group contents. In this treatment, the HOO–, which is produced by H_2_O_2_ under alkaline conditions, reacts with the quinone-methide intermediates that are formed by ionization of the phenolic lignin moieties or by alkali-induced cleavage of the cyclic α-aryl ether bonds in phenolic lignin moieties.^24^ The quinone-methide intermediates (see Fig. S2 in ESI†) are finally oxidized into dicarboxylic acids by the cleavage of the benzene ring in KL, resulting in the reduction of Ph-OH group content in KL.^25,26^ The treatment conditions and the properties of treated samples are listed in Table 2. After the treatment, the solution was neutralized using 1.0 mol L^−1^ H_2_SO_4_ and dialyzed for 48 h using the aforementioned membrane dialysis. The sample collected from membrane was dried and considered as peroxide-treated KL in this study.

**Table 2:** Hydrogen peroxide treatment conditions and Ph-OH content of peroxide-treated lignin

Sample	Lignin concentration, wt%	H_2_O_2_, wt%, based on lignin	Time, h	Temperature, °C	Carboxylate group, mmol g^−1^	Ph-OH, mmol g^−1^
1	5	18	1	90	1.65	0.74
2	5	8	1	80	1.06	0.95
3	5	4	1	80	0.65	1.23
4	5	4	0.5	80	0.43	1.39
KL	—	—	—	—	0.37	1.73

#### Polymerization of treated lignins with AA

The polymerization conditions of acetylated KL, methylated KLs and peroxide-treated KLs with AA were fixed at pH 3.5, lignin concentration 0.15 mol L^−1^, AA/lignin molar ratio 8.0, 80 °C, 3 h and 1.5 wt% initiator. After polymerization, the polymer was purified according to the procedure stated earlier. The carboxylate group content was measured according to the method detailed in the following section, and the increased carboxyl group of lignin was determined (*via* subtracting the carboxylate group of lignin–AA polymer from the total carboxylate group of KL).

#### Presence of unreacted kraft lignin in the polymerization

As stated previously, the unreacted KL may exist in the final lignin–AA polymer after the reaction. In order to evaluate this, acetone was used to extract the unreacted lignin from lignin–AA polymer samples extracted from the reaction medium in soxhlet extractor for 12 h. In this case, the unreacted lignin can be extracted from the lignin–AA polymer as kraft lignin is soluble in acetone, but lignin–AA polymer is insoluble.^27,28^ Initially, a certain amount (about 2.0–2.5 g) of sample, *M*_0_, was maintained in a pre-extracted filter paper, and then the samples were extracted with acetone for 12 h. After the extraction, the sample was air-dried and then dried in an oven at 105 °C for 12 h. The final mass of the sample, *M*_1_, was weighed and the percentage of unreacted lignin was calculated using eqn (3):3
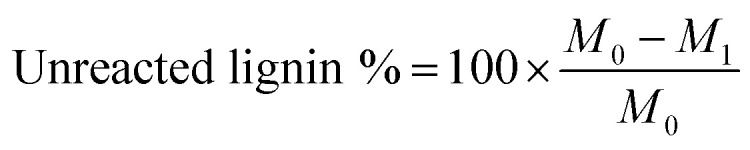


#### Functional group analysis

The carboxylate group and Ph-OH contents of lignin and lignin–AA polymers were measured using an automatic potentiometric titrator (785 DMP Titrino, Metrohm, Switzerland). About 0.06 g of dried KL or lignin–AA polymer, *m*, was added to 100 mL of deionized water containing 1 mL of 0.8 mol L^−1^ potassium hydroxide in a 250 mL beaker. After stirring at 200 rpm for 5 min, 4 mL of 0.5% *para*-hydroxybenzoic acid solution was added as an internal standard, and the solution was titrated with 0.1 mol L^−1^ hydrochloric acid solution. During the titration, with the decrease in the pH of the sample solutions, three endpoints appeared in sequence (
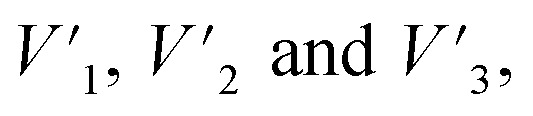
 respectively). The corresponding three endpoints in the titration curve of blank sample were specified as *V*_1_, *V*_2_ and *V*_3_, respectively. The carboxylate group and Ph-OH contents of samples were calculated according to eqn (4) and (5).^29^ The reported data in this paper is the average of three repetitions.4
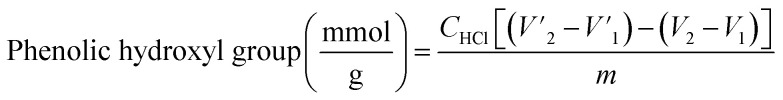
5
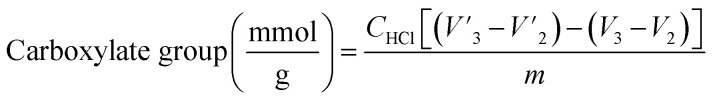
where *C*_HCl_ is the concentration of HCl solution (0.1 mmol L^−1^) as titrant, *m* is the mass (g) of the sample.

#### Molecular weight analysis

The molecular weight of samples was determined using a Gel Permeation Chromatography system, Malvern GPCmax VE2001 Module + Viscotek TDA305 with multi-detectors (UV, RI, viscometer, low angle and right-angle laser detectors). For KL measurement, the columns of PAS106M, PAS103 and PAS102.5 were used with a fixed flow rate of tetrahydrofuran (THF) at 1.0 mL min^−1^. For lignin–AA polymer measurement, the columns of PAA206 and PAA203 were used with a fixed flow rate of 0.1 mol L^−1^ NaNO_3_ solution at 0.70 mL min^−1^. The column temperature was set at 35 °C in both systems. Polystyrene polymers were used as standards for organic system and poly (ethylene oxide) for the aqueous system. Meanwhile, an attempt was made to cleave the PAA chain from lignin–AA *via* treating lignin–AA polymer in different acidic environments so that the molecular weight of PAA chain attached to lignin can be identified. However, this experiment was unsuccessful as the lignin part of lignin–AA polymer was also decomposed under acidic conditions prohibiting this analysis.

About 100 mg of air dried KL sample was initially suspended in 4.0 mL of acetic anhydride/pyridine 1/1 (v/v) solution by stirring for 30 min at 300 rpm and 25 °C; and then the solution containing KL was kept in a dark place at 25 °C for 24 h to acetylate KL. The resulting solution was then poured in an excess amount (50 mL) of ice water and centrifuged/washed 3 times. Afterwards, the solvent was removed from the samples using a freeze dryer. The acetylated KL was dissolved in 10 mL of tetrahydrofuran (THF) by stirring at 300 rpm for 30 min at room temperature, and then filtered with a PTFE filter having a diameter of 13 mm and pore size of 0.2 μm. The filtered samples were used for a molecular weight analysis. For lignin–AA polymer analysis, about 50 mg of air dried polymer samples was dissolved in 10 mL of 0.1 mol L^−1^ NaNO_3_ solution and filtered with a 0.2 μm nylon filter (13 mm diameter). The filtered solutions were used for molecular weight analysis.

#### Hydrodynamic diameter of lignin–AA polymer

The hydrodynamic diameters of lignin–AA polymers were measured using a dynamic light scattering instrument (type BI-200SM Brookhaven Instruments Corp., USA). The light source was a solid state laser with a maximum power of 35 mW and a wavelength of 637 nm. The experimental procedure was adopted as described by Yan *et al.*^30^ The lignin–AA polymer was dissolved in 1 mg mL^−1^ NaCl solution at pH 10.5 to make 0.2 wt% polymer concentration. The obtained solution was stirred for 30 min at 300 rpm and 25 °C. Then, the solution was kept for 24 h without stirring to have a well-dissolved polymer in the solution. After that, the sample solution of 20 mL was filtered with a 0.45 μm disposable syringe filter prior to the size measurement. Five measurements were performed for each sample and the mean value was reported. The analysis was conducted at 25 ± 0.02 °C. The scattering angle was set at 90°.

#### Elemental analysis

The elemental analysis was performed for KL and lignin–AA polymer using Elementar Vario EL Cube Elemental Analyzer by a method described in the literature.^31^ The samples were firstly dried in the oven at 105 °C overnight in order to remove any moisture. Approximately, 2 mg of samples were used for determining carbon, hydrogen and oxygen contents of the samples.

#### Fourier transform infrared (FTIR)

Fourier transform infrared spectroscopy (FTIR) analysis was conducted on KL and lignin–AA polymers. The samples were firstly dried in the oven at 105 °C overnight and 0.05 g of the samples was used for analysis using FTIR (Bruker Tensor 37, Germany, ATR accessory). The spectra were recorded in a transmittance mode in the range 600 cm^−1^ and 4000 cm^−1^ with 4 cm^−1^ resolution, and 32 scans per sample were conducted.

#### ^1^H-NMR analysis

The KL and lignin–AA polymer were analyzed with ^1^H-NMR analysis. The samples of dried KL and lignin–AA polymer were dissolved into D_2_O with 10.2 pH at a 40–50 g L^−1^ concentration. The solution was stirred for 30 min to fully dissolve the materials. The NMR spectra of these samples were recorded using an INOVA-500 MHz instrument (Varian, USA) with a 45° pulse and relaxation delay time of 1.0 s.

#### Solubility analysis of KL and lignin–AA polymer

The solubility of KL and lignin–AA polymer was determined based on the method described by Lappan *et al.*^32^ About 0.5 g of KL or lignin–AA polymer was added to 50 mL of deionized water at different pHs using 1.0 mol L^−1^ NaOH or 1.0 mol L^−1^ H_2_SO_4_ solution in a 125 mL Erlenmeyer flask. The suspension was immersed into a water bath shaker (Innova 3100, Brunswick Scientific, Edison, NJ, USA) and shaken (100 rpm) at 30 °C for 2 h. Then, the suspension was centrifuged at 1000 rpm for 5 min. The supernatants were collected and dried at 105 °C, which helped determine the solubility of KL and lignin–AA polymer in water at different pHs. To determine the solubility of KL or lignin–AA polymer, the mass of NaOH or H_2_SO_4_ added for adjusting the pH was taken into account.

#### Zeta potential and flocculation of aluminium oxide suspension

The zeta potential of aluminium oxide suspensions was analyzed (2.5 wt% at pH 6 and 8) *via* a zeta potential analyzer, ZetaPALS (Brookhaven Instruments Co., USA). In this study, 1 mL of the suspension was mixed with 20 mL of a 1 mM KCl solution. The zeta potential of the mixture was analyzed after 30 seconds of mixing. All the measurements were carried out three times at room temperature. The flocculation performance of lignin–AA polymer in an aluminium oxide suspension was evaluated by a photometric dispersion analyzer (PDA, PDA 3000, Rank Brothers, UK), which was connected to a dynamic drainage jar (DDJ). In this set of experiment, 450 mL deionized water with different pHs was firstly poured into the DDJ without any mesh. The system circulated water through PDA and DDJ for 10 min to reach a steady flow rate of 50 mL min^−1^. Then, 50 mL of a 2.5 wt% aluminium oxide suspension at different pHs was added to the DDJ while stirring at 100 rpm. The suspension was circulated in the system continuously at a flow rate of 50 mL min^−1^. After reaching a steady state condition, the lignin–AA polymer solution with 0.1 g L^−1^ concentration was added into DDJ to induce the flocculation process. The degree of flocculation was presented as a relative turbidity, which was calculated from the variations in the DC voltages of the PDA analyzer before and after adding lignin–AA polymer according to the eqn (6).^33,34^ The reported data in this paper is the average of three repetitions.6
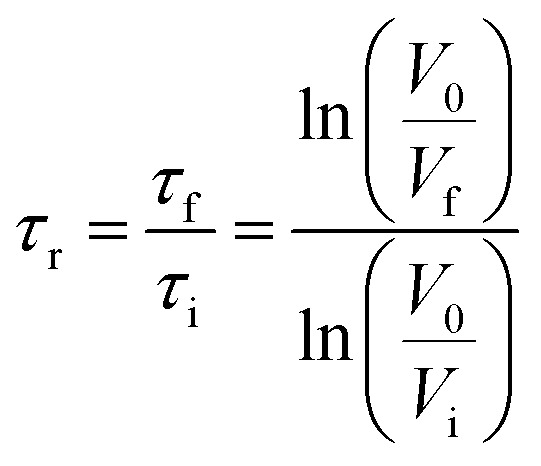
where *τ*_i_ is the initial turbidity of the aluminium oxide suspension (before adding lignin–AA polymer); *τ*_f_ is the final turbidity of aluminium oxide suspension (after adding lignin–AA polymer); *V*_0_ is initial base DC voltage (water solution); *V*_i_ stands for the DC voltage of aluminium oxide suspension (without lignin–AA polymer); and *V*_f_ is the DC voltage of the aluminium oxide suspension after adding lignin–AA polymer.

## Results and discussions

### Reaction mechanism of polymerization of KL and AA

#### Proposed reaction scheme

The free radical polymerization at low pH is usually suppressed, because the efficiency of homolytic scission of K_2_S_2_O_8_ in acidic conditions is considered lower than that in alkaline and neutral conditions.^35^ To investigate the influence of lignin on initiator decomposition, the final concentrations of the initiator under acidic and alkaline conditions was investigated when treated at 80 °C after 3 hours with an initial K_2_S_2_O_8_ concentration of 0.75 g L^−1^. It was discovered that the consumption of K_2_S_2_O_8_ under acidic conditions and alkaline conditions was similar in absence of lignin (*i.e.*, concentration of unreacted K_2_S_2_O_8_ was 0.40 g L^−1^ in acidic *vs.* 0.37 g L^−1^ in alkaline solutions). However, the unreacted K_2_S_2_O_8_ was 0.22 g L^−1^ in acidic and 0.72 g L^−1^ in alkaline solution in the presence of lignin, indicating that lignin probably inhibited the consumption of K_2_S_2_O_8_ under alkaline conditions. Thus, more radicals were produced at low pH by the redox systems in the presence of lignin, promoting polymerization under acidic conditions. The results in this study indicated that the mechanism of generation of free radicals was not solely attributed to the production of two sulfate radicals from the initiator. The redox system of persulfate-phenolic moieties of lignin could be also involved and affected by pH.^36^ The self-decomposition of potassium persulfate in the initiator system generate sulfate radicals.^37^ Sulfate radicals can remove hydrogen from the hydroxyl groups on lignin and generate lignin macro radicals. Once the macromolecular radicals are generated, they react with the monomers and initiate the polymerization.^38,39^

**Fig. 1:**
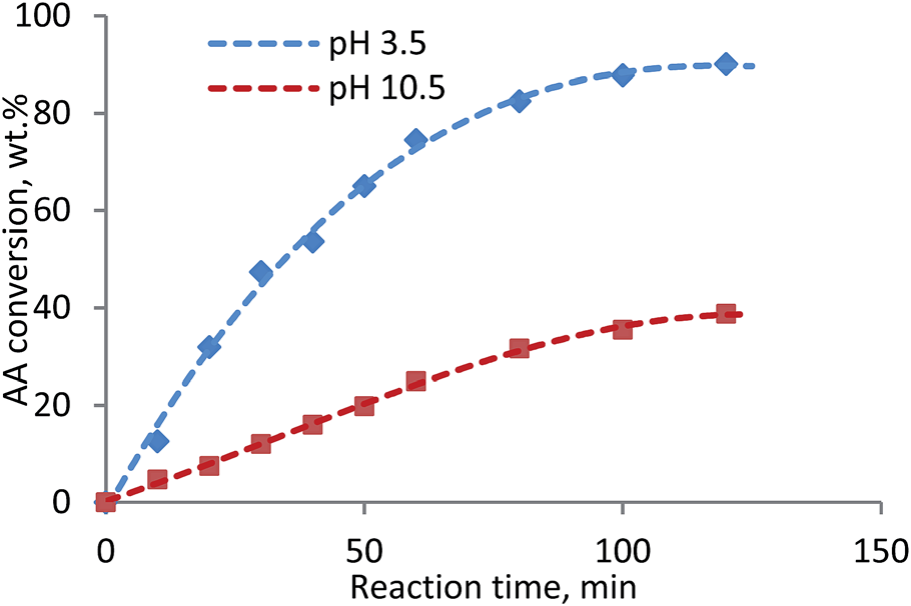
AA conversion in to produce PAA at pH 3.5 and 10.5 as function of reaction time.

The final KL–AA polymer from acidic and alkaline systems has very different charge density (1.86 meq g^−1^ under alkaline *vs.* 7.22 meq g^−1^ under acidic condition) and molecular weight (0.46 × 10^5^ g mol^−1^ under alkaline *vs.* 7.4 × 10^5^ g mol^−1^ under acidic condition). The AA conversion of AA homopolymerization system at pH 3.5 and 10.5 was measured and presented in Fig. 1. The AA conversion of reaction at pH 3.5 reached 90.1% in 2 h, however, it was only 38.8% at pH 10.5. Also, the molecular weights of PAAs from the reaction conducted at pH 3.5 and pH 10.5 were determined to be 4.26 × 10^5^ g mol^−1^ and 0.83 × 10^5^ g mol^−1^, respectively. This phenomenon was also observed in another work.^35^ One can conclude that the higher charge density and molecular weight of KL–AA polymer, prepared under acidic condition (pH 3.5), could be due to the fact that lignin accelerated the radical formation and thus more AA conversion.

In this study, the polymerization of KL and AA was carried out in an acidic aqueous suspension through heterogeneous reaction. To clarify if KL and PAA react *via* esterification, the PAA prepared in this study was used for investigating the reaction between KL and PAA with and without an initiator. The ^1^H-NMR analysis of the products of KL and PAA reaction, which is available in ESI (Fig. S3†), showed that no PAA was grafted onto KL; illustrating that (i) the esterification reaction between carboxylate groups of PAA and aliphatic hydroxyl groups of KL did not occur in the acidic system, and that (ii) the terminated PAA formed during the polymerization reaction of KL and AA cannot be reinitiated to form KL–AA polymers.

It was reported that the polymerization of styrene and hydrochloric acid lignin did not occur on aliphatic group of lignin molecules.^19,40^ In our previous work, we described that the polymerization did not occur on those groups in alkaline homogenous reaction of KL and AA.^21^ Due to the lowest bond dissociation energies of C_6_H_5_O–H (89.8 kcal mol^−1^), and C_6_H_5_CH_2_–H (90 kcal mol^−1^) among chemical groups in KL,^41,42^ the predominant lignin radicals, which are induced by the initiator radicals SO_4_^2−^˙ through the homolytic rupture of a bond in KL during acidic polymerization reaction, are phenoxyl radicals from phenolic lignin units, and with smaller probability, benzylic radicals from non-phenolic lignin units. It was also reported in the literature^42^ that acetylated lignin models had significantly lower free radicals (benzylic radicals) than the untreated lignin samples. To clarify whether the polymerization of KL and AA occurred through the benzylic radicals in the acidic conditions, the acetylated KL was used to polymerize with AA. The final product was analyzed using ^1^H-NMR (Fig. S4 in ESI†) and showed that the characteristic peaks of PAA did not exist on the product of acetylated KL and AA; demonstrating that (i) the polymerization of AA onto KL through benzylic radicals was not detectable in acidic system, (ii) in the absence of phenolic hydroxyl group, the aromatic ring, methoxyl group and aliphatic portion of lignin molecules did not participate in the reaction, and (iii) in the absence of phenolic hydroxyl group in KL, the polymerization of AA and KL was not noticed in acidic conditions.

**Fig. 2:**
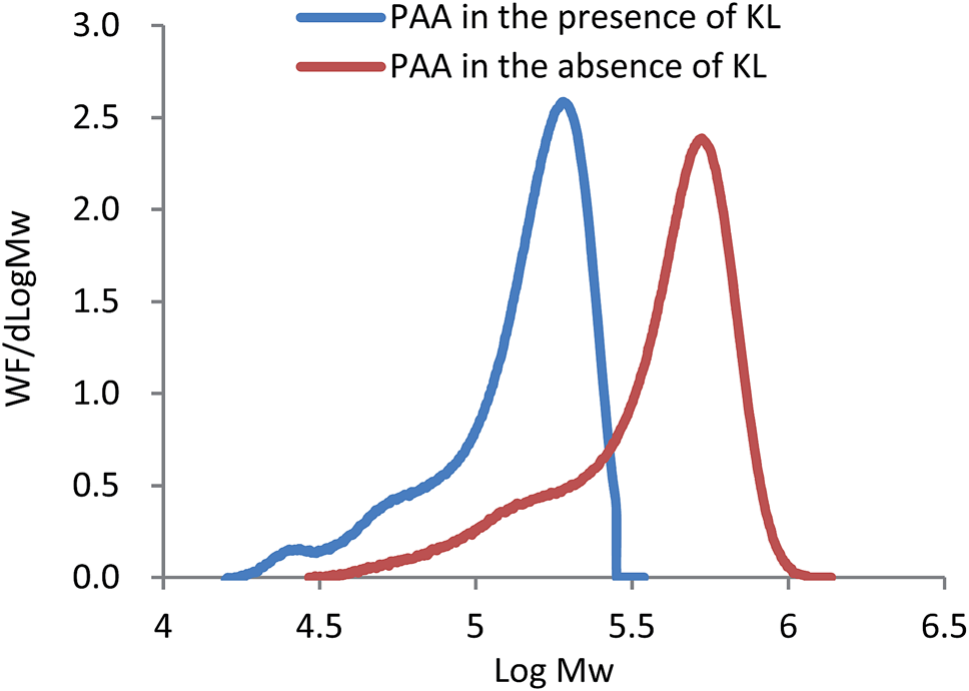
The distribution of molecular weight of PAA from KL–AA in the presence and absence of KL.

In addition to the lignin radicals formed by the initiator, the chain transfer reactions of KL and the growing PAA radicals may form lignin radicals. To testify if KL functions as a radical transfer in this system, the molecular weight of PAA formed in the absence and presence of KL was measured and shown in Fig. 2.

**Fig. 3:**
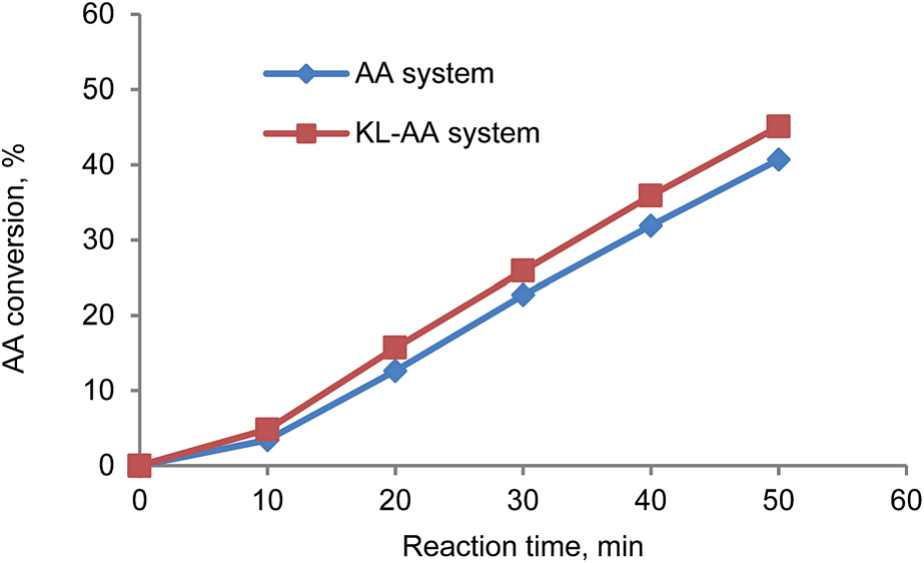
AA conversion in KL–AA system and AA system as function of reaction time.

As presented in Fig. 2, the molecular weight of PAA in the presence of KL (111 700 g mol^−1^) was much lower than that in the absence of KL (426 300 g mol^−1^), illustrating that (i) KL functioned as a chain transfer agent in this polymerization system, and (ii) the chain transfer reaction between KL and PAA chain radicals formed some lignin radicals for this polymerization. As it is well known, the chain transfer in polymerization system not only results in a reduced molecular weight of the polymer, but may also affect the polymerization rate, which depends on the reinitiation reaction rate between chain transfer radicals and monomers. In order to understand the effect of KL on the polymerization rate, the AA conversion in KL–AA polymerization system and AA homopolymerization system was determined and shown in Fig. 3. It is apparent that KL slightly increased the AA conversion, illustrating that the reinitiation reaction rate between lignin radicals and monomers is similar or slightly higher than that of propagation reaction of AA chain radicals, which is also consistent with the findings in the polymerization of lignosulfonate with AA in the literature.^43^ The reason for this behaviour can be ascribed to a greater decomposition of the initiator in the presence of KL (as discussed earlier).

**Scheme 1:**
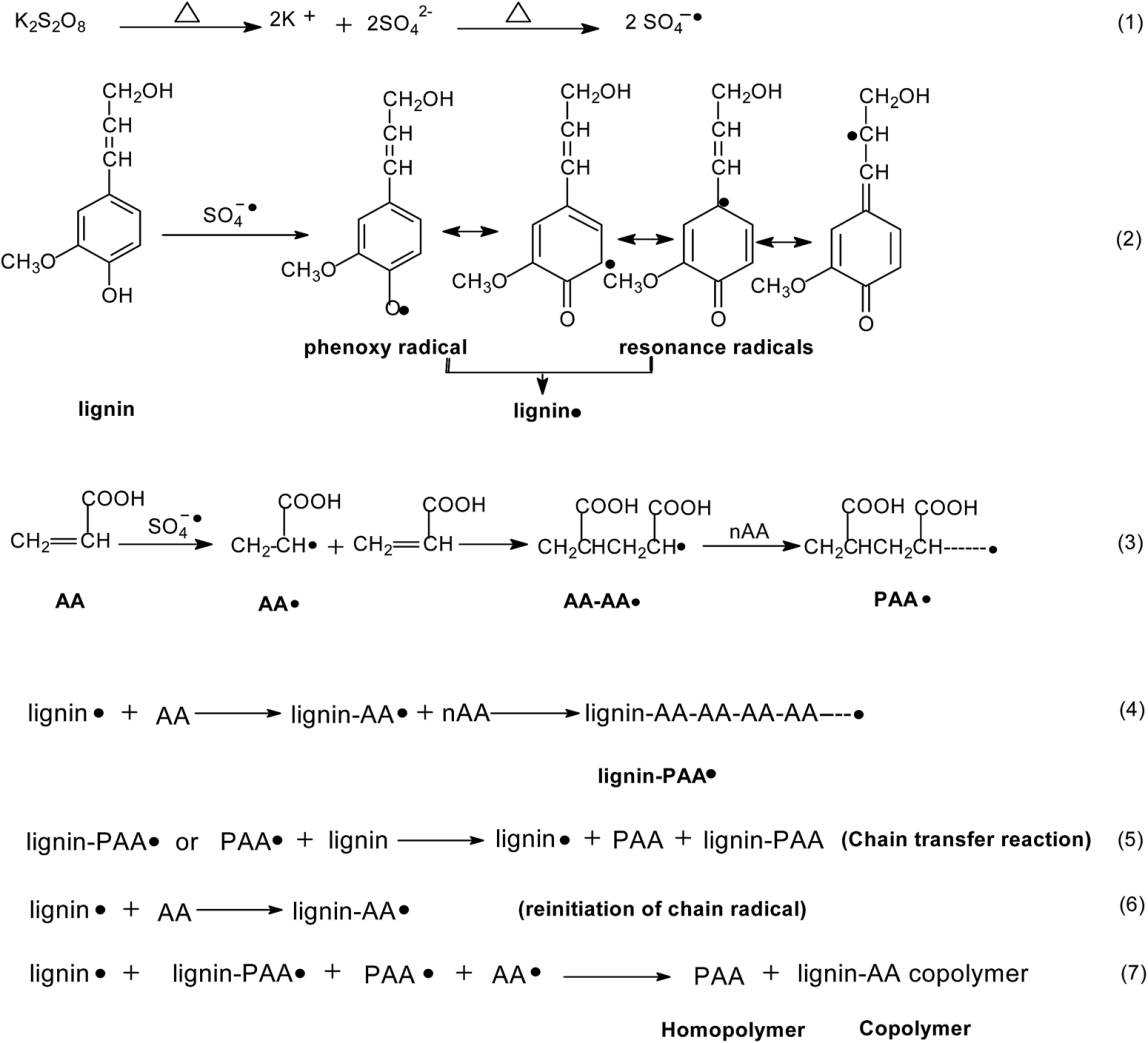
Proposed reaction scheme of polymerization of KL and AA initiated by K_2_S_2_O_8_ under acidic conditions.

Based on the analysis above, the proposed reaction scheme of KL and AA polymerization is shown in Scheme 1. As softwood KL is known to be composed principally of coniferyl alcohol units,^44^ it was chosen to present KL in this scheme. In this polymerization reaction, the sulfate radicals can initially be formed by heat decomposition (reaction (1)), which then react with phenolic hydroxyl group of KL to generate phenoxy radicals and its resonance radicals (reaction (2)). The formation of the KL radicals will lead to more decomposition in reaction (1) and more sulfate radicals. Also, the sulfate radicals can initiate AA to form AA radicals (AA˙). The AA radicals then react with other AA monomers to form PAA chain radicals (reaction (3)).^9,11^ The KL radicals (lignin˙) react with monomer (AA) to form propagated lignin–AA chain radicals (reaction (4)). In addition, the PAA chain radicals and lignin–AA radicals in the system can react with KL to form KL radicals through a chain radical transfer reaction (reaction (5)), and the KL radicals then reinitiate AA to form lignin–AA chain radicals (reaction (6)). Finally, the propagated AA chain radicals and propagated lignin–AA chain radicals react with each other to produce PAA homopolymer and lignin–AA polymer through termination reaction as shown in reactions (7). As a result, the carboxylate groups are introduced onto KL and the molecular weight of KL is increased.

#### Impact of Ph-OH group on the KL–AA polymerization

As shown in Scheme 1, the OH group on the phenolic structure of KL was converted to ether or carbonyl structures in the final polymer product, which would reduce the Ph-OH content of the polymer. To clarify this, the Ph-OH contents of KL before and after treating with initiator or polymerization reaction were measured. The Ph-OH content of KL was originally 1.73 mmol g^−1^, but it decreased to 1.59 mmol g^−1^ after treating with K_2_S_2_O_8_. After polymerization with AA, the Ph-OH content of KL was further decreased to 0.55 mmol g^−1^. One can conclude that the Ph-OH content in KL declined during the polymerization, which was mainly attributed to the participation of Ph-OH in the polymerization.

**Fig. 4:**
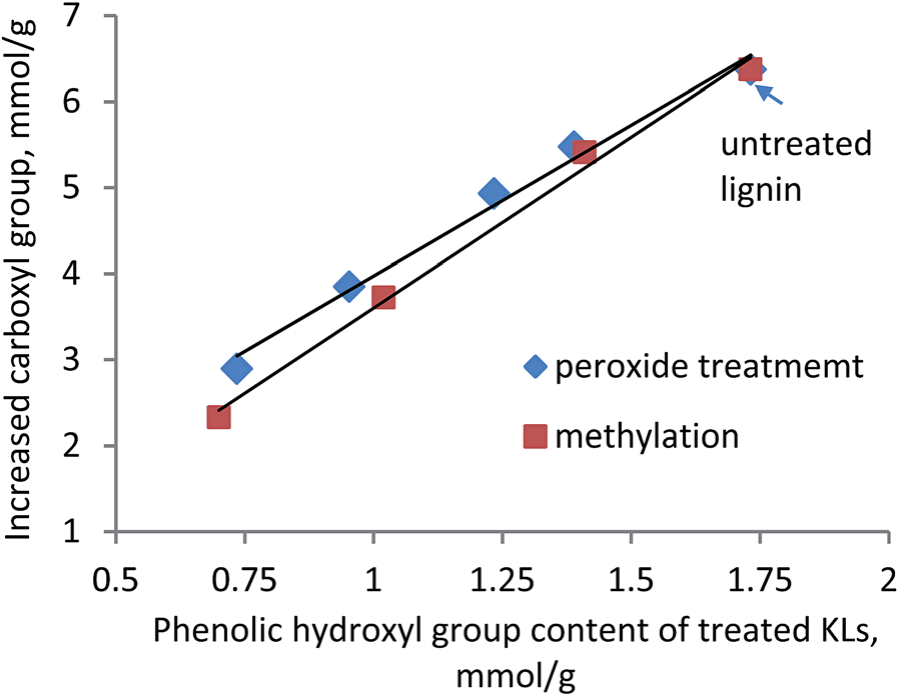
Relationship between Ph-OH group of KL and increased carboxylate group of KL–AA polymer.

In another set of experiments, the impact of Ph-OH content of KL on the polymerization efficiency was determined *via* treating KL with hydrogen peroxide, which can reduce the Ph-OH content of lignin.^45,46^ The H_2_O_2_ treatment conditions and the Ph-OH content of the treated lignin are listed in Table 2. As seen in Table 2, by increasing the dosage of H_2_O_2_, the temperature and time of the treatment, the Ph-OH content of the resulting KLs decreased; whereas, the carboxylate content of the treated KLs increased, which was due to the oxidation of KL by H_2_O_2_.^46,47^ The treated and untreated KLs were polymerized with AA, and the carboxylate group of the resulting lignin–AA polymer was measured. Fig. 4 presents the impact of Ph-OH of KL on carboxylate content of KL–AA polymer. It can be seen that the Ph-OH content of lignin has a linear relationship with the increased carboxylate group of the final polymer, indicating that the OH group attached to the phenolic structure of KL is the reaction site for the polymerization.

To further assess the relationship between phenolic hydroxyl group of KL and increased carboxyl group of KL–AA polymer, the methylated lignins with different amounts of phenolic group were polymerized with AA. The increased carboxylate group had a linear relationship with the phenolic hydroxyl group of KL (Fig. 4), demonstrating the importance of phenolic hydroxyl group of KL on polymerization. It is also seen in this figure that, at a high amount of phenolic hydroxyl group on either peroxide-treated lignin or methylated lignin, the increased carboxylate groups for lignin–AA polymers are similar. However, with decreasing the amount of the phenolic hydroxyl group, the increased carboxylate group of the resulting polymer was more pronounced for peroxide-treated KL.

The reasons for this phenomenon might be (i) the more open structure of lignin after peroxide treatment compared with that after methylation, and (ii) the lower molecular weight and higher solubility of peroxide-treated KL compared with methylated lignin. In other words, the more open structure provides higher accessibility of AA to the reaction sites on KL. To clarify this, the acetylated peroxide-treated lignin was reacted with AA and the final product was analyzed using ^1^H-NMR (Fig. S5† in ESI†). The results showed that AA was not polymerized with the acetylated peroxide-treated lignin, illustrating the fact that there were no other reaction sites on the peroxide-treated lignin. Therefore, it can be concluded that the more significant increase in the carboxylate group of peroxide-treated lignin–AA polymer was attributed to the easier accessibility of AA to the phenolic hydroxyl group of KL, as the reaction site for the polymerization was still phenolic group of peroxide treated KL. This phenomenon was also observed by Phillips *et al.*^19^ in the polymerization of styrene and calcium lignosulfonate.

#### Participation of KL in polymerization

To investigate the participation of lignin in this heterogeneous polymerization with AA, acetone was used as a solvent to extract unreacted KL (if present) from the lignin–AA polymer after the polymerization, since KL has a high solubility in acetone but lignin–AA polymer was insoluble in acetone solution. The results of this analysis are listed in Table 3. In the absence of AA in the reaction, the percentage of unreacted lignin reached 98.8%, demonstrating that the lignin's properties were not affected either by the initiator treatment or the acidic treatment. In the absence of the initiator, the percentage of the unreacted lignin was also higher than 99%, which could be regarded as evidence that basically no reaction occurred. However, in the presence of AA, initiator and KL in the polymerization, the amount of unreacted lignin was minimal (only 0.61–1.13 wt%). Furthermore, the reaction was very fast as the amount of unreacted lignin was marginal even after 0.5 h of the reaction. One can conclude from the analysis that almost all of the KL participated in the polymerization with AA in acidic conditions.

**Table 3:** Reaction conditions and percentage of unreacted KL^a^

Sample	AA/lignin molar ratio, *n* : 1	Time, h	Initiator, wt% on lignin mass	Percentage of unreacted lignin, %
1	1 : 1	0.5	1.5	1.07
2	1 : 1	2	1.5	1.13
3	0.5 : 1	2	1.5	1.08
4	2 : 1	2	1.5	0.61
5	1 : 1	2	0	99.3
6	0 : 1	2	1.5	98.8
7	0 : 1	2	0	99.5

a Other reaction condition: temperature 80 °C, lignin concentration 0.15 mol L^−1^, pH 3.5.

### Effects of reaction conditions

#### Initiator

**Fig. 5:**
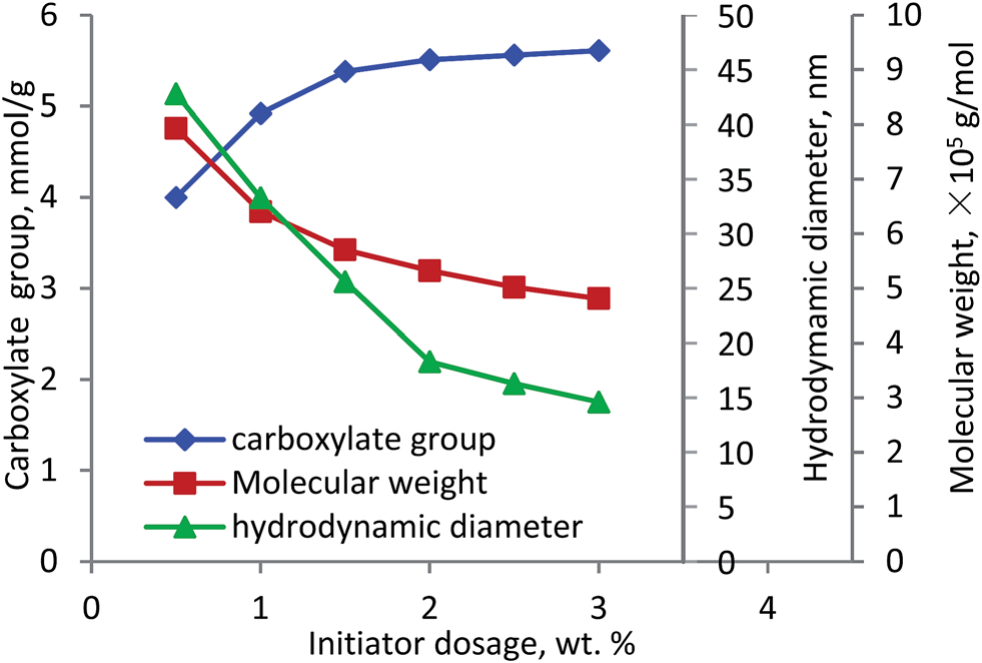
Carboxylate group content, molecular weight and hydrodynamic diameter of lignin–AA polymer as a function of initiator dosage (pH 3.5, lignin concentration 0.15 mol L^−1^, AA/lignin molar ratio 5.5, 80 °C, 3 h).

The effect of initiator dosage on the carboxylate group content and molecular weight of lignin–AA polymer was investigated and the results are shown in Fig. 5. With the increase in the initiator dosage from 0.5 wt% to 1.5 wt%, the carboxylate group content increased from 4.0 mmol g^−1^ to 5.38 mmol g^−1^. Further increase in the dosage marginally increased the carboxylate group content. However, the molecular weight of lignin–AA polymer decreased from 7.9 × 10^5^ g mol^−1^ to 4.8 × 10^5^ g mol^−1^ when the dosage of the initiator increased from 0.5 to 3.0 wt%. It was well known that the lower the dosage of the initiator, the fewer the radicals (grafting cites) would form on lignin, which would result in a polymer with a longer chain.^48,49^ The polymerization of lignin and AA can occur under two possibilities (i) a small number of long PAA segment attached to lignin in final KL–AA polymer or (ii) a large number of short PAA segment attached to lignin in final KL–AA polymer. In the former case, the hydrodynamic diameter of the polymer would increase due to the presence of long PAA segment on lignin. In the latter case, as a large number of short PAA segment would be grafted on lignin, it may not change the hydrodynamic diameter of lignin significantly. To clarify this, the hydrodynamic diameters of lignin–AA polymers produced were measured *via* using different initiator dosages and presented in Fig. 5. With the increase in the initiator dosage, the hydrodynamic diameter of the polymer decreased, illustrating the polymer produced at high initiator dosage probably had more of shorter PAA segment. It should be noted that, the hydrodynamic diameters of KL were 6.1 nm. The distributions of hydrodynamic diameter of KL and KL–AA polymer are provided in Fig. S6 in ESI† and the results depicted better uniformity for KL–AA polymer than for KL in the solution.

#### Reaction time

**Fig. 6:**
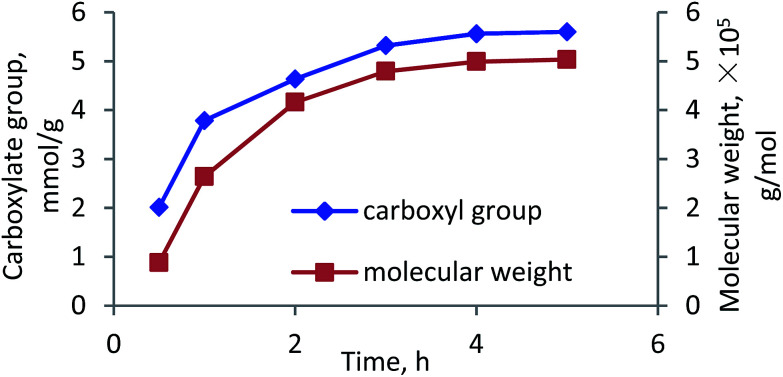
Carboxylate group content and molecular weight of lignin–AA polymer as function of reaction time (pH, 3.5, initiator, 1.5 wt%, lignin concentration, 0.15 mol L^−1^, AA/lignin molar ratio, 5.5, 80 °C).

The effects of reaction time on the carboxylate group and molecular weight of lignin–AA polymer are shown in Fig. 6. It can be seen that both carboxylate group and molecular weight increased with extending the reaction time. The carboxylate group and molecular weight of lignin–AA polymer significantly increased from 2 meq g^−1^ to 5.32 meq g^−1^ and from 1.0 × 10^5^ to 5.0 × 10^5^ g mol^−1^, respectively. The increases in the carboxylate group and molecular weight could be attributed to the addition of monomers to the growing of grafted chains at an extended reaction time.^50,51^

#### AA/lignin molar ratio

**Fig. 7:**
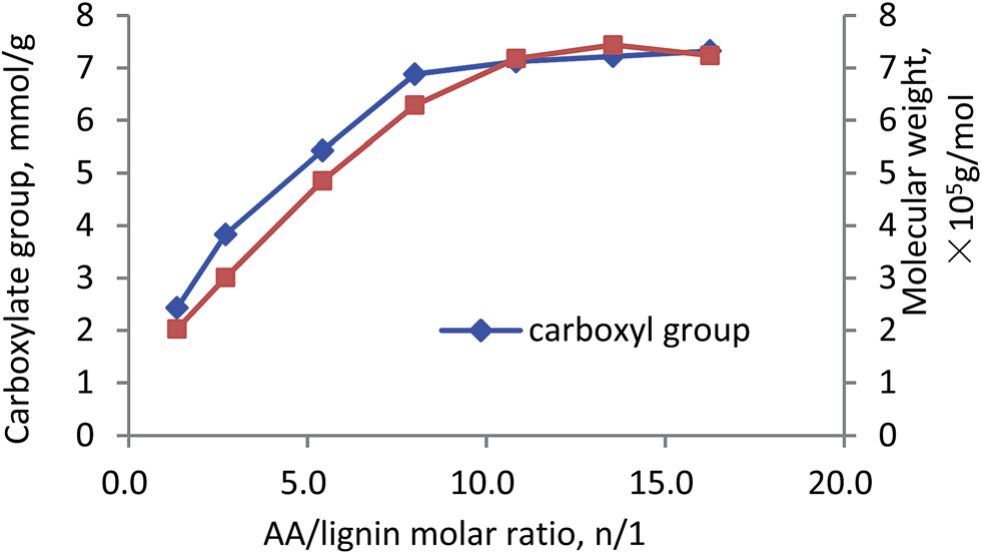
Carboxylate group content and molecular weight of lignin–AA polymer as a function of AA/lignin molar ratio (pH, 3.5, initiator, 1.5 wt%, lignin concentration, 0.15 mol L^−1^, 3 h, 80 °C).

The influence of AA/lignin molar ratio on the carboxylate group and molecular weight of the polymer is shown in Fig. 7. It can be observed that by changing AA/lignin molar ratio from 1.4 to 10.8, the carboxylate group and molecular weight increased rapidly to 7 meq g^−1^ and 7.1 × 10^5^ g mol^−1^, respectively. Further increase in the ratio marginally affected the charge density and molecular weight of the polymer. The increases in the carboxylate group and molecular weight showed an acceleration in the polymerization rate of lignin and AA due to an increased AA content in the reaction medium.^52^ However, when the ratio of AA/KL was higher than 13.5, the homopolymerization of AA to generate PAA was probably dominated that decelerated the polymerization of KL and AA (carboxylate group and *M*_w_ were constant).^50,53^

#### Temperature

**Fig. 8:**
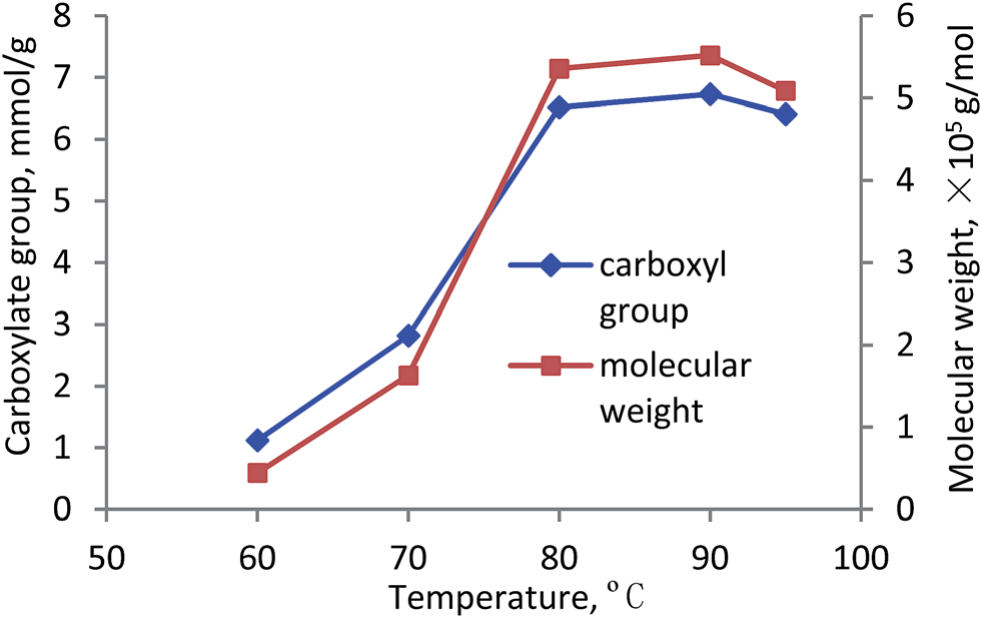
Carboxylate group content and molecular weight of lignin–AA polymer as a function of temperature (pH, 3.5, initiator, 1.5 wt%, lignin concentration, 0.15 mol L^−1^, AA/lignin molar ratio, 8.0, 3 h).

The effects of reaction temperature on the carboxylate group and molecular weight of lignin–AA polymer are presented in Fig. 8. It can be seen that with rising the reaction temperature from 60 °C to 80 °C, the carboxylate group and molecular weight of lignin–AA polymer increased dramatically from 1.12 mmol g^−1^ and 0.4 × 10^5^ g mol^−1^ to 6.52 mmol g^−1^ and 5.4 × 10^5^ g mol^−1^, respectively. The increases in the carboxylate group and molecular weight are attributed to the more effective access of AA monomer to the reaction sites on the lignin at a higher temperature, which could be due to the extended conformation of lignin molecules and the dissociation of lignin from its self-assembly of aggregates at a high temperature.^54,55^ In addition, the results in Fig. 8 suggests that the polymerization of KL with AA is endothermic reaction as it was promoted at a higher temperature. When the temperature was higher than 90 °C, both carboxylate group and molecular weight decreased, which was due to the fact that the higher temperature made the initiator less effective.^4,9^ Also, a high temperature favored the chain termination and chain transfer reactions as well as the competing homopolymerization (PAA) reaction.^10,28,43,52^

#### Lignin concentration

**Fig. 9:**
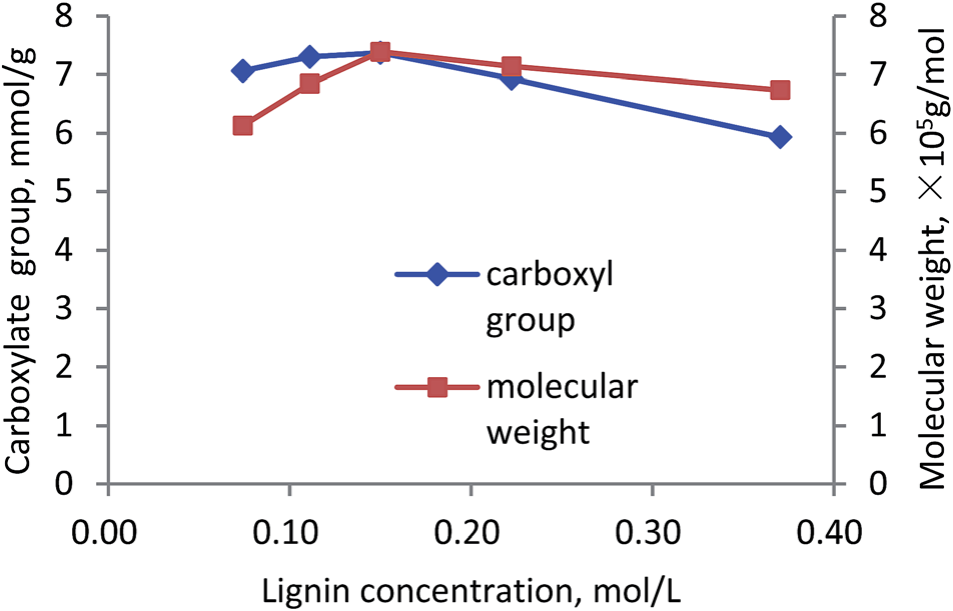
Carboxylate group content and molecular weight of lignin–AA polymer as a function of lignin concentration (pH, 3.5, initiator, 1.5 wt%, AA/lignin molar ratio, 10.0, 3 h, 80 °C).

The effects of lignin concentration on carboxylate group and molecular weight of lignin–AA polymer are shown in Fig. 9. As is observed, by concentrating lignin in the solution, the carboxylate group and molecular weight of lignin–AA polymer increased to 7.37 mmol g^−1^ and 7.4 × 10^5^ g mol^−1^ at 0.15 mol L^−1^ lignin concentration, which are attributed to the increased amount of phenolic hydroxyl radicals as well as increased probability of collision among lignin radicals, monomer radicals and the initiator molecules to form lignin–AA polymer.^56–58^ When lignin concentration was higher than 0.15 mol L^−1^, the phenolic hydroxyl radicals had more chances to interact with other lignin radicals, such as benzyl or phenolic hydroxyl radicals, by disproportionation or radical coupling reactions.^4,50^ Thus, the active radicals, capable of initiating the polymerization of monomers, were ineffectively consumed, leading to the declined molecular weight and carboxylate group.

**Fig. 10:**
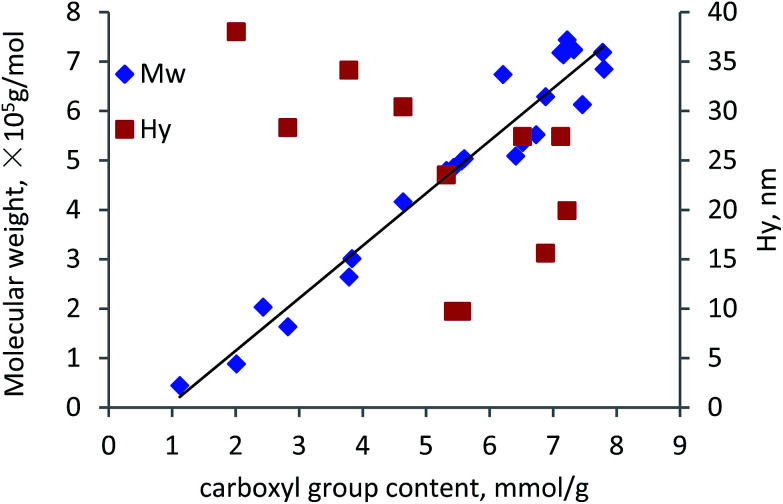
Relationship between carboxylate group content of lignin–AA polymer with its molecular weight (based on 1.5 wt% initiator).

To investigate the relationship between carboxylate group and molecular weight of lignin–AA polymer, the data presented in previous figures is plotted in Fig. 10. As seen, the molecular weight of the polymer increased linearly with the increase in the carboxylate group content, indicating that the increase in the molecular weight of the polymer was mainly attributed to the PAA segment in the KL–AA polymer. The linear relationship between carboxylate group content and molecular weight is *Y* = 1.0618*X* − 0.9738, *R*^2^ = 0.9462, where *Y* is the molecular weight (×10^5^ g mol^−1^) and *X* is the carboxylate group of KL–AA polymer (mmol g^−1^). This formula can be used to correlate the molecular weight of lignin–AA polymer with its carboxylate group content. In addition to the molecular weight, the *H*_y_ of lignin–AA polymers with different carboxylate group contents for the same samples were measured and presented in Fig. 10. The *H*_y_ of lignin–AA polymers did not show a linear relationship with the carboxylate group, illustrating that even though lignin–AA with a determined molecular weight can be generated, the formed lignin–AA polymer may have different molecular conformations (coiled/linear) in solutions if they were produced under different conditions. The higher *H*_y_ of samples with lower carboxylate group may imply that, when the amount of carboxylate group (and molecular weight) of polymer was low, the polymer was linear or more lignin was involved, but with high carboxylate group (and high MW) the polymer generated a coil shape conformation.

Based on the results shown in previous figures, the optimal conditions for producing lignin–AA polymer were 0.15 mol L^−1^ KL, AA/KL molar ratio 10.0, initiator 1.5 wt% (based on lignin mass), 80 °C and 3 h. Under these conditions, the carboxylate group and molecular weight of KL–AA polymer reached 7.37 mmol g^−1^ and 7.4 × 10^5^ g mol^−1^, respectively. This lignin–AA polymer was selected for further analysis.

### Characterization of lignin–AA polymer

#### FTIR

**Fig. 11:**
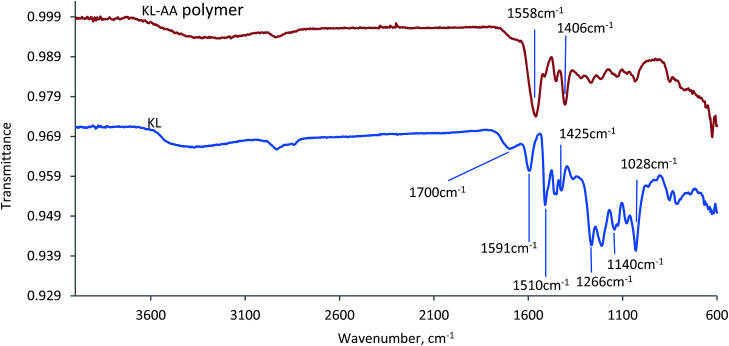
The FTIR spectra of KL–AA polymers and KL.

The FTIR spectra of KL–AA polymer prepared under the optimal conditions and KLs are presented in Fig. 11. Both KL and KL–AA polymer showed a broad band around 3400 cm^−1^, which is assigned to the OH stretching of phenolic and aliphatic compounds, and a band around at 2900 cm^−1^, which is assigned to the C–H stretching in the methyl groups.^59–61^ The absorption band at 1700 cm^−1^ in the spectra of KL and KL–AA polymer is assigned to carbonyl groups conjugated with an aromatic ring.^62^ In the spectrum of KL, two absorption bands were observed at around 1266 cm^−1^ and 1140 cm^−1^, which are assigned to C–O stretch of guaiacyl unit and C–H stretch of guaiacyl unit, respectively, illustrating that KL was a softwood lignin.^63^ The characteristic bands for the aromatic skeletal vibration of KL were located at around 1591, 1510 and 1425 cm^−1^, respectively.^63^ In the spectrum of KL–AA polymer, two new strong absorption peaks appeared at 1558 cm^−1^ and 1406 cm^−1^, which were absent in the spectrum of KL. These two peaks belong to carboxylic acid and symmetrical stretching vibrations of carboxyl anions –COO^−^, which illustrates the existence of PAA chain segment in the KL–AA polymer.^64,65^ The absorption peaks at 1510 cm^−1^ and 1425 cm^−1^ in the spectrum of KL–AA polymer, which were assigned to aromatic skeletal vibration of KL and demonstrated the existence of aromatic ring of KL,^43^ was evident for successful polymerization of KL and AA. More interestingly, the results in Fig. 11 show that the relative intensity of the band at 1028 cm^−1^, which belongs to non-etherified Ph-OH groups in KL–AA polymer, was weaker than that in KL, suggesting that lignin participated in the polymerization reaction through its active phenolic hydroxyl groups.^64^

#### Elemental analysis

The elemental analysis of KL and KL–AA polymer was carried out and the results are tabulated in Table 4. It is clear that the oxygen content of KL–AA polymer was increased from 27.04% in KL to 31.69 wt% in the polymer. Moreover, with the increase in the carboxylate group and the molecular weight, the oxygen content increased, while the carbon and hydrogen contents decreased. These changes on the carbon, hydrogen and oxygen contents of lignin in the polymer (compared to KL) were mainly because of the presence of PAA chains on lignin–AA polymer. In another report on the polymerization of amylopectin and AA, the hydrogen and oxygen contents of amylopectin were increased from 6.15% and 46.86% to 7.25% and 54.27%, respectively, but its carbon content decreased from 46.99% to 38.48%.^48^

**Table 4:** Elemental analyses of lignin–AA polymer

Sample	Carbon, wt%	Hydrogen, wt%	Oxygen, wt%	Formula	Carboxylate group, mmol g^−1^	*M*_w_, × 10^5^ g mol^−1^
KL	62.6	5.69	27.04	C_9_H_9.60_O_2.85_	0.37	0.17
KL–AA	45.3	3.92	31.69	C_9_H_9.34_O_4.72_	7.37	7.4

#### ^1^H-NMR analysis

**Fig. 12:**
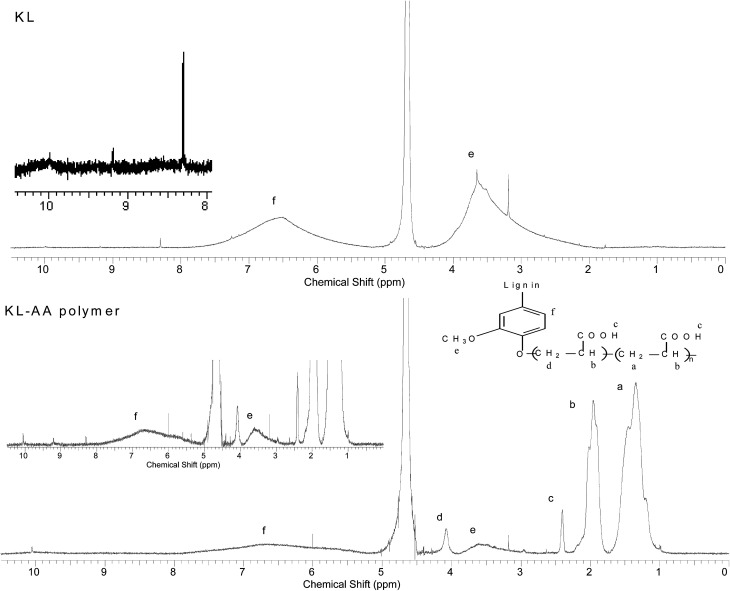
H-NMR spectra of KL and KL–AA polymer.

The ^1^H-NMR spectra of KL and KL–AA polymer are shown in Fig. 12, respectively. In Fig. 12, the peak at 9.97 ppm is attributed to the carboxylate group in KL; at 9.20 ppm is attributed to the hydrogen linked to the aldehyde group; at 8.30 ppm is associated with unsubstituted phenolic protons; at 7.42–5.99 ppm is attributed to aromatic protons (f on the figure). Moreover, the peak at 5.75–5.15 ppm is attributed to aliphatic protons including H_α_ and H_β_; at 3.9–2.55 ppm is associated with protons in methoxyl groups (e on the figure) of lignin; and at 3.20 ppm is assigned to the methylene protons in β–β structure.^66–68^ Peaks appeared at 4.5–4.9 ppm are assigned to the solvent of D_2_O. In Fig. 12, it can be observed that the peaks for PAA chain segment appeared at 1.4 ppm, 2.0 ppm and 2.4 ppm, respectively. Peaks appeared at 1.4 ppm is attributed to C-1 (a), at 2.0 ppm is attributed to C-2 (b), and 2.4 ppm is assigned to carboxylic acid end hydrogen of PAA (c).^28^ The peaks at 3.20 ppm, 2.55–3.0 ppm, 5.15–5.75 ppm, 5.99–7.42 ppm, 8.30 ppm and 9.2 ppm belonging to KL illustrate the successful polymerization of KL and AA. In addition, the peak at 4.10 ppm is observed in the spectrum of KL–AA polymer, which is absent in that of KL and assigned to the protons of –CH_2_– (d in the figure) connected with aromatic structure through ester bond (–CH_2_–O–C_6_H_5_).^69,70^ This also confirms that the Ph-OH groups are the active sites participating in the polymerization reaction, which is consistent with the FTIR results. The decrease in the peak intensity at 8.30 ppm, assigned to unsubstituted phenolic group of KL in the KL–AA polymer, demonstrated a decline in the residual Ph-OH group content of the polymer, which is consistent with the results discussed in the previous section.

#### Water solubility of KL–AA polymer

**Fig. 13:**
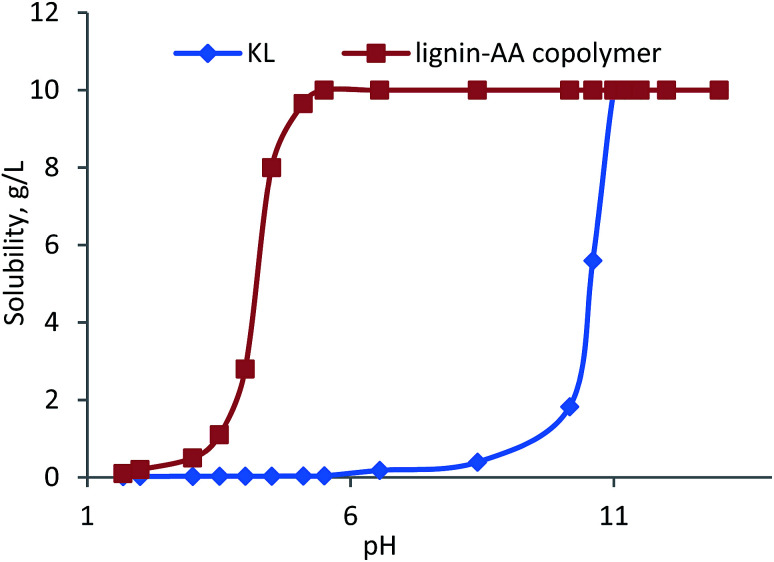
The solubility of KL–AA polymer and KL as a function of pH at 10 g L^−1^ concentration.

The solubility of KL–AA polymer and KL was presented as a function of pH in Fig. 13. As can be seen, at pH 10, the solubility of KL dropped dramatically to less than 2 g L^−1^. Interestingly, KL–AA polymer was soluble under acidic conditions to pH 4, below which KL–AA polymer became insoluble. As is well known, the p*K*_a_ of carboxylic acid is around 4.75, implying that the solubility of KL–AA polymer is due to the presence of carboxylate groups at a pH higher than 4.0.^71^ Also, it was observed that at pH 7, KL had a very low solubility (only 0.2 g L^−1^); however, KL–AA polymer had 10 g L^−1^ solubility, which illustrated the dramatic increase in the solubility of KL *via* this polymerization. Here, it should be noted that the highest concentration of KL–AA polymer water (in pH 7) can be as high as 100 g L^−1^.

### Flocculation performance of KL–AA polymer for aluminium oxide suspension

**Fig. 14:**
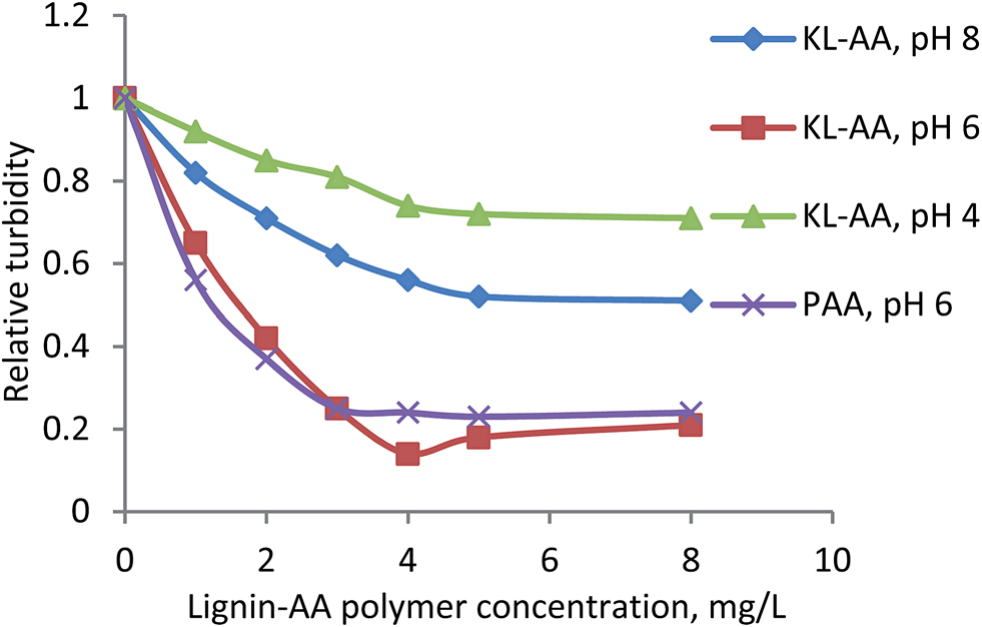
Flocculation performance of KL–AA polymer at different concentrations and pHs for aluminium oxide suspension.

Aluminum oxide is an industrially important oxide mineral. The flocculation of aluminum oxide particles is a key step for treatment of wastewater from mining industry.^72^ The flocculation characteristics of KL–AA polymer at different pHs were assessed in a 2.5 wt% aluminum oxide suspension, and the results are presented in Fig. 14. With an increase in the concentration, the flocculation efficiency of KL–AA was enhanced, but better results were obtained for KL–AA polymer at pH 6. As reported in the literature, flocculation can be promoted *via* charge neutralization, bridging, and hydrophobic/hydrophobic interaction.^73,74^ The reasons for better flocculation efficiency of KL–AA polymer at pH 6 are due to the fact that the (1) surface charge of aluminum oxide particles is positive at pH 6 (zeta potential, 12.4 mV), which can be neutralized by the negative charge of KL–AA polymer and (2) the KL segment of the KL–AA polymer offers the hydrophobic/hydrophobic interaction with aluminum oxide.^73^

Furthermore, at a low concentration (*e.g.*, 1 mg L^−1^ concentration), the flocculation efficiency of PAA is higher than that of KL–AA polymer. This is attributed to the amount of charges introduced to the particles. The carboxyl group content of PAA (*i.e.*, 10.2 mmol g^−1^) is much higher than that of KL–AA polymer (*i.e.*, 7.4 mmol g^−1^). At the same concentration, PAA offers more negative charges than KL–AA polymer to the surface of particles, providing a more neutralization degree to aluminum oxide particles and thus a higher flocculation efficiency. At 3 mg L^−1^ concentration for PAA and 4 mg L^−1^ concentration for KL–AA polymer, the amount of charges introduced to particles are similar, *i.e.*, 29.5 mmol L^−1^ with KL–AA polymer *vs.* 30.6 mmol L^−1^ with PAA, inducing a similar turbidity for aluminum oxide suspensions. At higher dosages, a higher flocculation efficiency was achieved for KL–AA polymer than for PAA indicating that, in addition to the PAA segment in KL–AA polymer, the lignin segment in KL–AA polymer contributed to the flocculation (*i.e.*, a more important role of KL component than PAA charge). Furthermore, the higher molecular weight (7.4 × 10^5^ g mol^−1^) and higher *H*_y_ (25.2 nm) of KL–AA than those of PAA (4.26 × 10^5^ g mol^−1^ and 7.2 nm) might have played roles in flocculation at the optimum dosage.

## Conclusions

The polymerization mechanism of KL and AA under acidic conditions was comprehensively studied in this work. It was discovered that KL promoted the decomposition of the initiator under acidic conditions. Also, more PAA chain would be grafted on KL under acidic conditions than alkaline. The results suggested that the phenolic hydroxyl group content of KL had a significant influence on the AA polymerization. The optimal conditions for the polymerization were 0.15 mol L^−1^ KL, AA/KL ratio of 10.0 mol mol^−1^, 1.5 wt% initiator, 80 °C and 3 h. Under the optimized conditions, the carboxylate group content and the molecular weight of the KL–AA polymer were 7.22 meq g^−1^ and 7.4 × 10^5^ g mol^−1^, respectively. The FTIR, H-NMR and elemental analyses confirmed the successful polymerization of KL and AA. Additionally, the resulting KL–AA polymer was water soluble at a pH higher than 4.5 and its maximum solubility was 100 g L^−1^. Compared with PAA, the KL–AA polymer was a more efficient flocculant for aluminum oxide suspensions at pH 6.

## Conflicts of interest

There are no conflicts to declare.

## Supplementary Material

RA-008-C7RA12971H-s001
