# Supplementary material for: Novel pathway to produce high molecular weight kraft lignin–acrylic acid polymers in acidic suspension systems
Source: RSC Adv. 2018 Mar 29;8(22):12322–36. doi: 10.1039/c7ra12971h (PMC9079287; doi:10.1039/c7ra12971h)
Supplement: RA-008-C7RA12971H-s001 [file RA-008-C7RA12971H-s001.pdf]

Electronic Supplementary Material (ESI) for RSC Advances.
This journal is © The Royal Society of Chemistry 2018

Novel pathway to produce high molecular weight kraft lignin-acrylic acid polymers in acidic suspension systems

Fangong Kong[1,2], Shoujuan Wang[1,2*] Weijue Gao[2] and Pedram Fatehi*[2]

1. Key Laboratory of Pulp and Paper Science and Technology of Ministry of Education, China, Qilu University of technology, Jinan, China, 250353

2. Chemical Engineering Department, Lakehead University, Thunder Bay, ON, Canada, P7B 5E1

**corresponding authors, email: (P. Fatehi) pfatehi@lakeheadu.ca; Tel: 807-343-8697; (S. Wang): swang18@lakeheadu.ca*

Purifying lignin-AA copolymer

After completion, the temperature of the solution was cooled to room temperature by immersing the flasks in a water bath at 15 ℃ for 20 min, and then the pH of the solution was adjusted to 1.5 by adding sulfuric acid and deionized water, while stirring at 300 rpm, to make the final volume of the solution 200 mL. This treatment would lead to the precipitation of lignin-AA copolymer. The suspension was centrifuged at 4000 rpm for 10 min to collect the precipitated copolymer from the suspensions (PAA and unreacted AA monomers). This acidification/centrifugation was repeated 3 times for the complete removal of lignin-AA copolymer from AA monomer and PAA. Additionally, Genesys 10s UV-Vis spectrophotometer (Thermo Scientific, Madison, USA) was used to measure the absorbance at 280 nm to testify the absence of the lignin-AA copolymer in the acidic supernatants containing PAA homopolymer and AA monomer. The prepared PAA in this study with molecular weight of 440,000 g/mol were also mixed with kraft lignin without reaction, and then separated through the acidification/centrifugation process. After 3 cycles of acidification/centrifugation, there was no PAA in the precipitates, which was confirmed by H-NMR analysis, illustrating that the acidification/centrifugation process was effective in separating lignin-AA copolymer from PAA or unreacted AA. Subsequently, the precipitated lignin-AA copolymers were mixed with 100 mL deionized water. After adjusting the pH of the solution to 7.0 ± 0.2, the samples were dialyzed using the dialysis membrane for 48 h to remove the inorganic salt, which was induced to the solutions by pH adjustment. The deionized water used for dialysis was changed once every 4 h in the first 24 h and every 6 h in the following

24 h. After dialysis, the solution was dried at 105 °C, and the dried sample was considered as the final lignin-AA copolymer.

Purification of PAA from AA system and KL-AA system

For the AA system (in absence of KL), after the reaction, the solution was neutralized using 0.1 mol/L sulfuric acid and kept in membrane tubes to dialyze for 48 h to remove the remaining impurities (e.g. inorganic salt induced by pH adjustment) and unreacted AA from the PAA. The deionized water used for dialysis was changed once every 6 h. After dialysis, the solution was dried at 105 °C, and the dried sample was considered as prepared PAA in absence of KL.

For the KL-AA system, upon completion of reaction, the solution was treated according to the procedure stated above. The supernatant of this separation process was collected and neutralized using sodium hydroxide, and then dialyzed for 48 h to remove the unreacted AA and inorganic salt. Then, the sample was dried at 105 °C, and the dried sample was considered as prepared PAA in presence of KL.

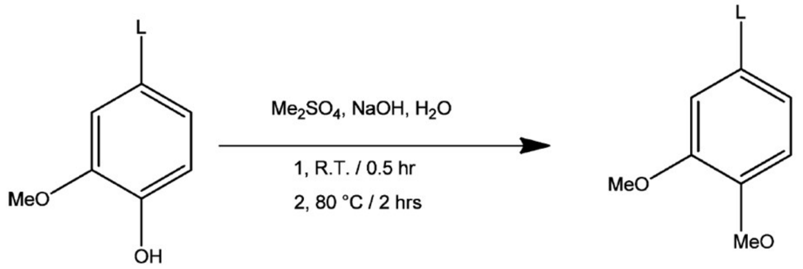

Figure S1 Methylation of KL by using dimethyl sulfate in aqueous NaOH [23] (Me-CH3, Me_2SO_4-dimethyl sulphate)

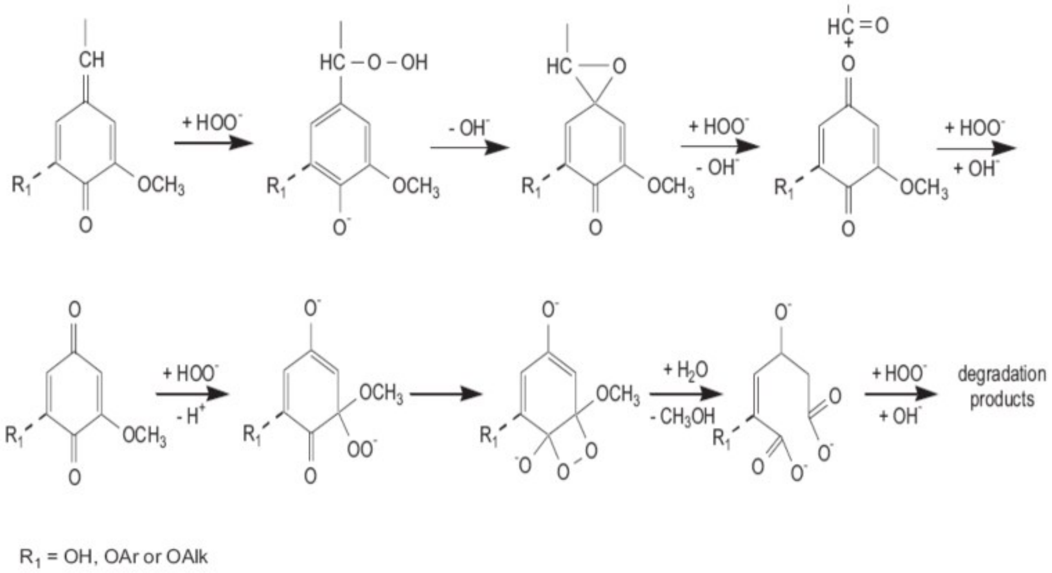

Figure S2 Reaction scheme of KL with H_2O_2 [25, 26]

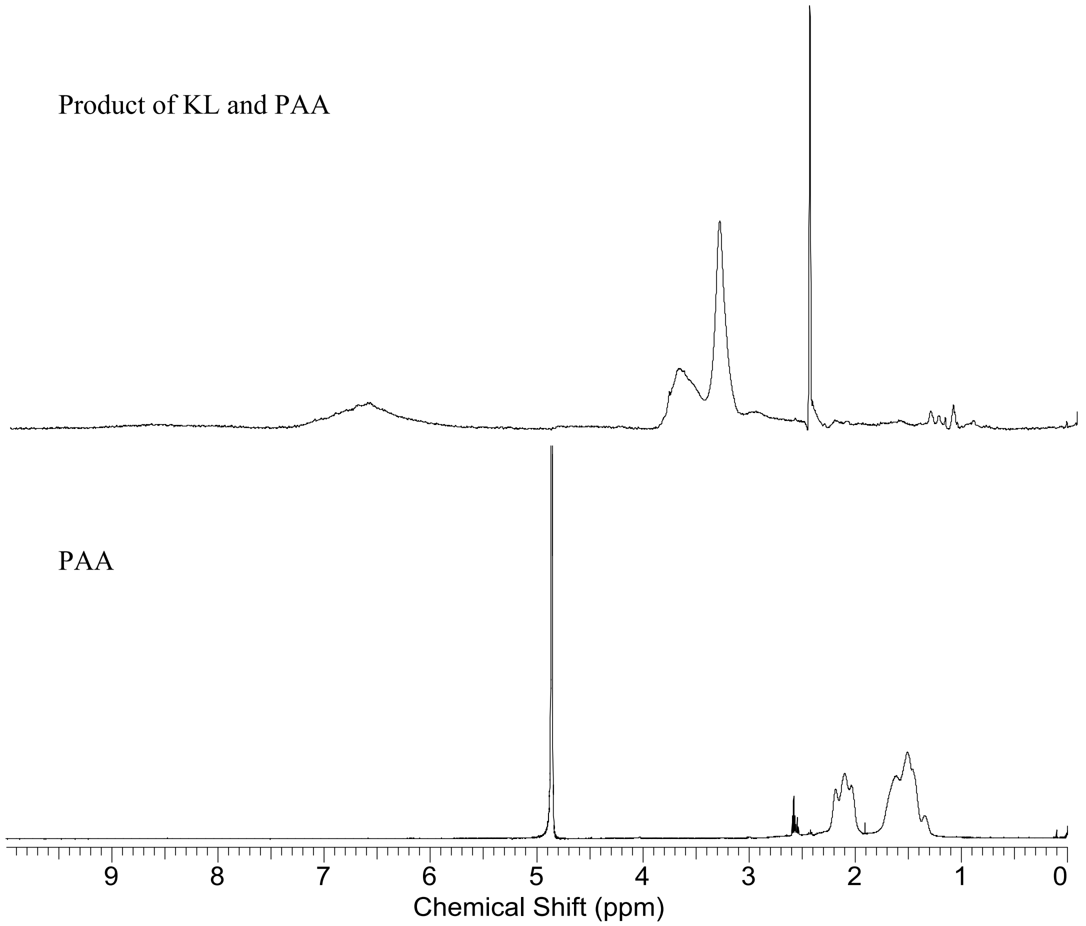

Figure S3. H-NMR spectra of product of KL and PAA, and PAA

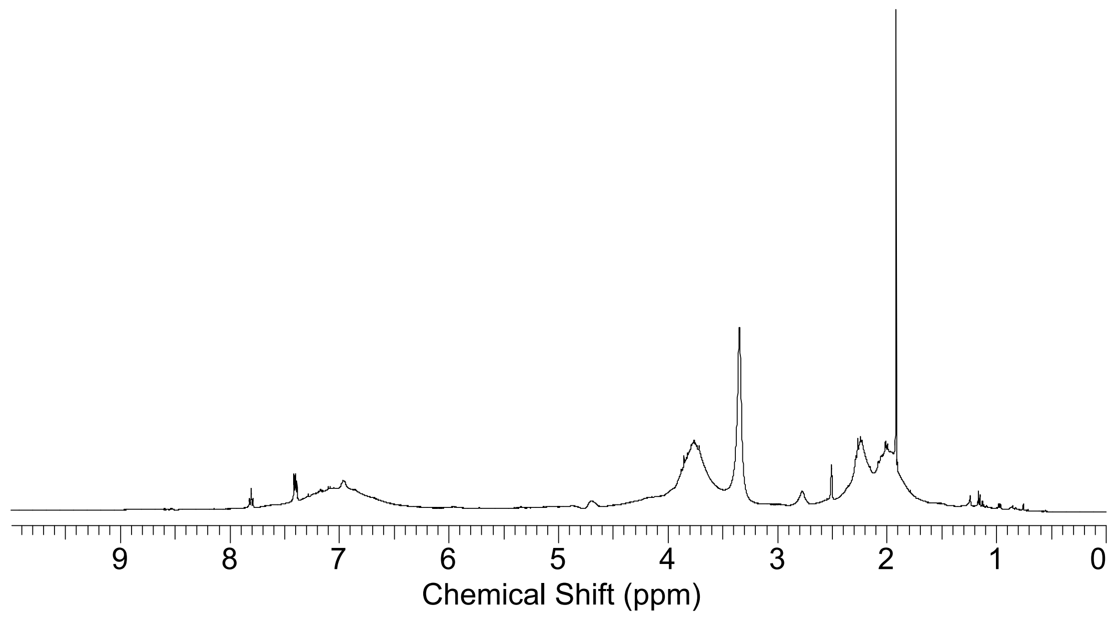

Figure S4. H-NMR spectrum of product of acetylated KL and AA

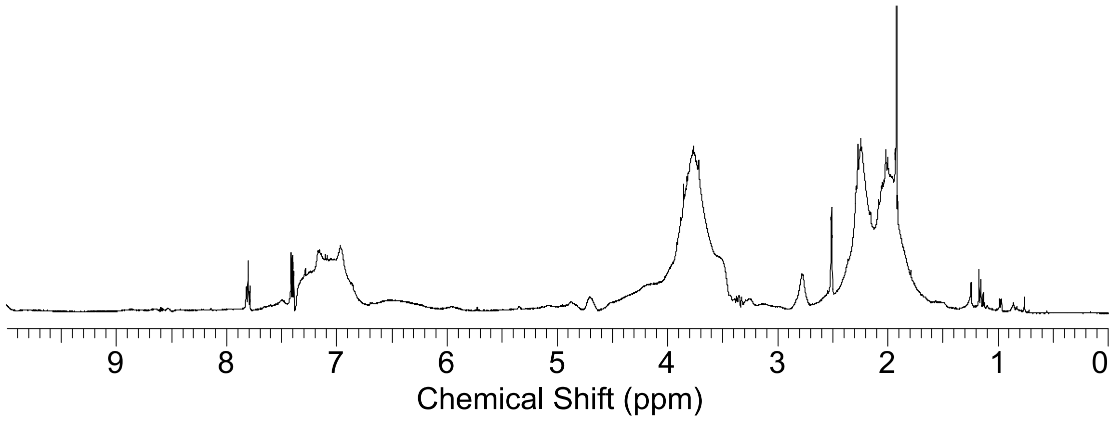

Figure S5. H-NMR spectrum of product of acetylated peroxide-treated KL and AA

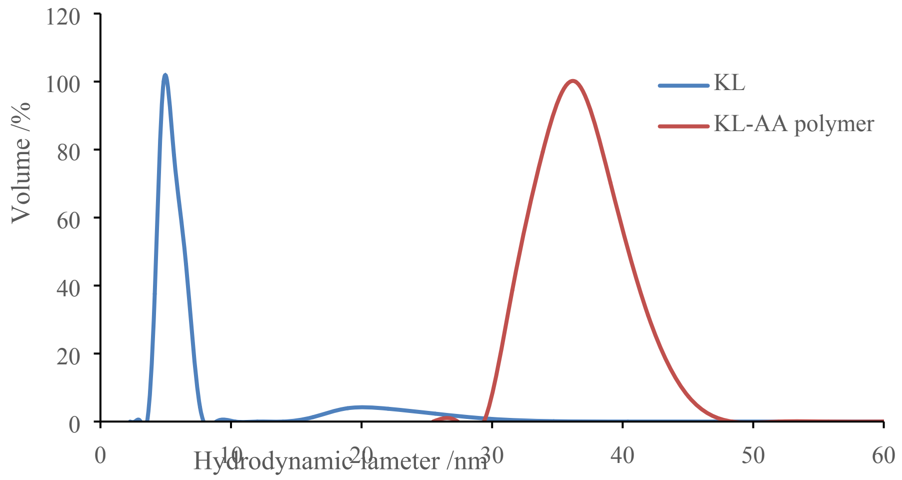

Figure S6 Distribution of hydrodynamic diameter of KL and KL-AA polymer in solution
